# Are different generations of CAD/CAM milling machines capable to produce restorations with similar quality?

**DOI:** 10.4317/jced.52984

**Published:** 2016-10-01

**Authors:** Renato Roperto, Hussein Assaf, Thiago Soares-Porto, Lisa Lang, Sorin Teich

**Affiliations:** 1 DDS, MS, PhD, Department of Comprehensive Care, School of Dental Medicine – Case Western Reserve University, 2124 Cornell Rd, Cleveland, 44106, USA; 2 DDS, MS, Department of Comprehensive Care, School of Dental Medicine – Case Western Reserve University, 2124 Cornell Rd, Cleveland, 44106, USA; 3 DDS, MS, MBA, Department of Comprehensive Care, School of Dental Medicine – Case Western Reserve University, 2124 Cornell Rd, Cleveland, 44106, USA; 4 DDS, MBA, Department of Comprehensive Care, School of Dental Medicine – Case Western Reserve University, 2124 Cornell Rd, Cleveland, 44106, USA

## Abstract

**Background:**

Different CAD/CAM machines’ generation may impact the restoration overall quality. The present study evaluated the marginal fit of CAD/CAM restorations manufactured with different generations of CEREC milling unit systems.

**Material and Methods:**

Sixteen typodont teeth were divided into two groups (n=8) according to the machine’s generation assigned. These are control group (G1): Cerec AC with Bluecam/Cerec 3 milling unit and (G2): Cerec AC with Bluecam/MC XL Premium Package milling unit. Scanning of the preparation were performed and crowns were milled using the Vita Mark II blocks. Blocks were cemented using epoxy glue on the pulpal floor only and finger pressure applied for 1 min. Upon completion of the cementation step, misfits between the restoration and abutment were measured by microphotography and the silicone replica technique using light body silicon material on Mesial (M) and Distal (D) surfaces.

**Results:**

Mean and SDs of marginal gaps in micrometers were: G1/M: 94.90 (±38.52), G1/D: 88.53 (±44.87), G2/M: 85.65 (±29.89), G2/D: 95.28 (±28.13). Two-way ANOVA indicated no significant differences among different groups (*P*>0.05); surface area (*P*>0.05) and the interaction (*P*>0.05). Overall, G2 had greater margin gaps than G1, however, without statistical difference (*P*>0.05).

**Conclusions:**

Difference in milling unit generation did not significantly affect the marginal fit. Marginal gap means were in the range of the clinical acceptance levels for both generations of Cerec milling units, regardless the teeth site area.

** Key words:**CAD/CAM, margin, ceramics.

## Introduction

Cerec CAD/CAM machines are currently used to manufacture ceramic restorations based on computer-assisted design and produce a restoration on a single dental appointment. These restorations, commonly made with ceramic material, are becoming increasingly popular worldwide (1-4). Because the technology is costly, some clinicians are still using previous generations of CAD/CAM equipment to fabricate and delivery intraoral restorations.

Margin quality has been described as one of the most important aspects when comes to longevity of CAD/CAM ceramic restorations (5-12). Margin discrepancies beyond 100µm may impact the survival rate by causing microleakage, staining, tooth sensitivity, recurrent caries, periodontal problems, and ultimately failure of the entire restoration (13,14).

The present study was designed to compare and evaluate the marginal fit of ceramic CAD/CAM ceramic restorations produced with the Cerec AC using BlueCam and milled with different Cerec milling generations: i) Cerec 3 Milling unit (Cerec3) and ii) Cerec MC XL Premium Package (MCXLPP) milling unit. First, we determined whether the marginal fit would be affected by the different milling unit used, and second, we examined if the different regions of the tooth (mesial and distal) differ in terms of marginal fit, regardless of the milling unit used.

## Material and Methods

This study was approved by an ethics committee. Sixteen maxillary typodonts (Kilgore typodont model 200 – Kilgore International, Coldwater, MI, USA) with unrestored and intact teeth were used in this study. The upper left second pre-molars typodont teeth received a full-contour crown preparation for Cerec with 6-8o degrees of axial wall conversion, 1.0mm axial reduction, and a 2.0 mm flat occlusal reduction on both functional and non-functional cusps. A flat 90o shoulder margin design was used for all preparations.

**Figure 1:**
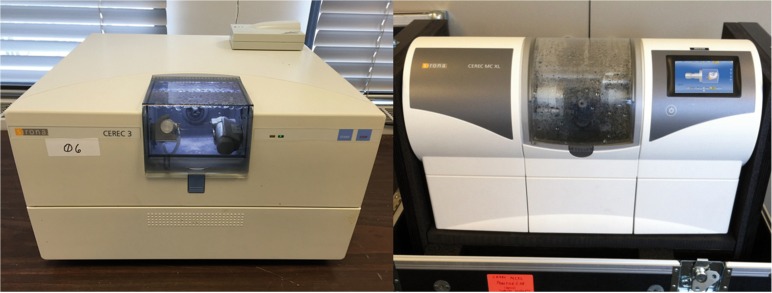
Milling CAD/CAM units used in this study: Cerec 3 (left) and Cerec MXCL Premium Package (right).

The preparations were made using cone-shape diamond points (016 FG medium round end taper diamond) followed by a finishing diamond point with the same shape (Brasseler, Savannah, GA, USA). The gingival margin was then finished with a flat end diamond point (014 FG medium flat end taper diamond - Brasseler), resulting in a 1.0mm width circumferential flat shoulder. All preparations had 0.5mm supragingival margins and they were prepared by one investigator. A new set of diamond points was used for every four prepared teeth. Teeth were then randomly assigned to two groups (n=8). These are control group (G1): Cerec 3 Milling unit (Cerec3) and ii) Cerec MC XL Premium Package milling unit (MCXLPP) (Fig. 1). Technical differences between these two machines are listed in  Table 1.

**Table 1:**
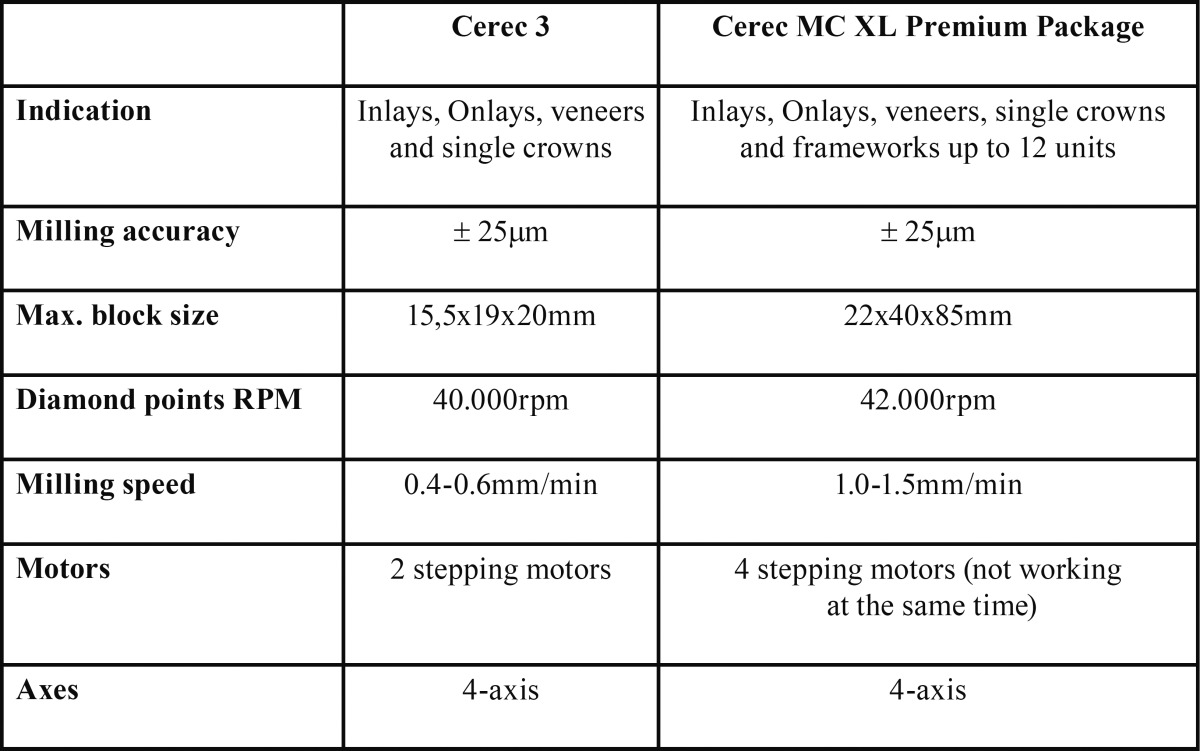
Cerec 3 and Cerec MCXL Premium Package comparative chart.

A Cerec AC unit with Bluecam (Sirona) equipped with Microsoft Windows 7-64 bits (Microsoft Corporation, Redmond, WA, USA) that run the Cerec software version 4.3 (Sirona) was used in this study. For each group of teeth, the intra-oral camera was calibrated using the Sirona camera calibration kit. An uniform layer of anti-reflective spray (Sirona Optspray) was sprayed on all teeth surface area, including the surrounding soft tissue area. Special care was taken to avoid over-powdering the prepped teeth and adjacent surfaces, which were then scanned with the intra-oral camera. Before generating the 3D model, scanned images for both G1 and G2 were evaluated on the computer screen using the “distance” software tool in order to confirm preparation metrics.

A tri-dimensional (3D) model was then generated for each tooth; the adjacent teeth were digitally set in the virtual model axis and trimmed for proximal contact adjustments. Preparation margin was designed using the “automatic margin finder” tool followed by the insertion axis adjustment. The Cerec software then generated the 3D proposal restoration using the following software crown parameters: spacer= 100 µm; occlusal milling offset= 0 µm; proximal contact strength= 50 µm; occlusal contact strength= -75 µm; dynamic contact strength= 25 µm; minimal thickness (radial)= 0 µm; minimal thickness (occlusal)= 0 µm; margin thickness= 70 µm. After generating the 3D virtual restorations, scanned images for both G1 and G2 were again evaluated on the computer screen using the “restoration thickness” tool in order to confirm restoration metrics.

**Figure 2:**
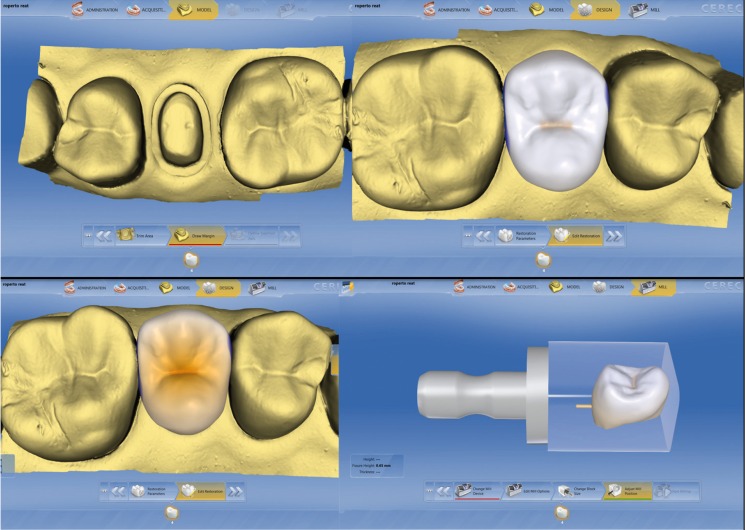
Sequence for tooth scanning (top left), crown design (top right), crown metrics adjustment (bottom left) and milling preview (bottom right).

The Cerec3 milling unit was calibrated using the Sirona Milling Calibration kit and a new set of diamond burs kit (step bur 12 and point bur 10) were used. One brand new water tank filled with distilled water and lubricant (Dentatec-Sirona) was used for the millings on this group. The MCXLPP was also calibrated by replacing the milling diamonds for the calibration phantom and pins. A brand new water tank with a brand new filter was used with distilled water mixed with lubricant (Dentatec-Sirona). The CAD/CAM feldsphatic ceramic blocks Vita Mark II (Vita, Yorba Linda, CA, USA) size I12 and 1M1 shade ceramic blocks were employed to mill all the restorations for both groups. Upon completion of each milling process, blocks were replaced inside the milling chamber and the diamond burs inspected for damage or breakage. Prepared teeth were then stored in small envelopes with their respective ceramic restorations according to the group assigned. The sequence for tooth scanning, crown design and milling preview is shown in figure 2.

**Figure 3:**
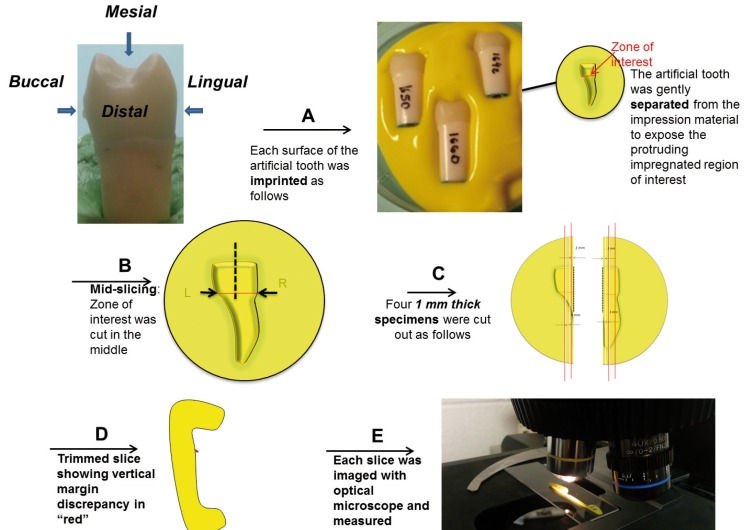
Silicon replica technique sequence.

Teeth were then submitted to the micrographic silicon replica technique for margin misfit evaluation. The silicon replica technique flow chart is shown in figure 3. Mesial and distal surfaces of each group were evaluated separately as follows. The light body silicon based polymer was pooled in a shallow container. The tooth specimen was gently positioned into the uncured, low viscosity polymeric mixture without full submergence and allowed to remain inside the polymer until the substrate was fully cured. Subsequently, the tooth was gently separated from the polymeric material in order to expose impression of the region of interest. The polymeric imprint of a particular side of the tooth was cut in the middle with a fresh razor blade and subsequently 1 mm thick slices were cut from the specimen to the right and to the left of the mid-section, yielding overall eight slices for analysis. Each slice was carefully marked to identify its position and viewed in a reflective optical 40x magnification microscope (Nikon SMZ445, Melville, NY, USA) to measure the variations in the marginal fit. A linear glass scale with 10µm resolution was used to identify the measured length in the specimens.

Data were analyzed by statistical software (SPSS 23.0, IBM Software, Armonk, NY, USA). Differences in the milling units among groups, also the individual sites, were analyzed by two-way ANOVA with Tukey’s post hoc test comparisons to find any statistically significant differences. The significance level was α=0.05.

## Results

Inspection of histograms, normal Q-Q plots, and box plots showed that the measurements for preparation type and each site individually are normally distributed. The mean and standard deviation (SD) associated with vertical gap (μm) related to different milling units are: Cerec3 milling 91.71±41.61 and MCXLPP 90.46±29.20. Among the sites; the two-way ANOVA analysis did not show statistical differences between both milling units ( Table 2).

**Table 2:**
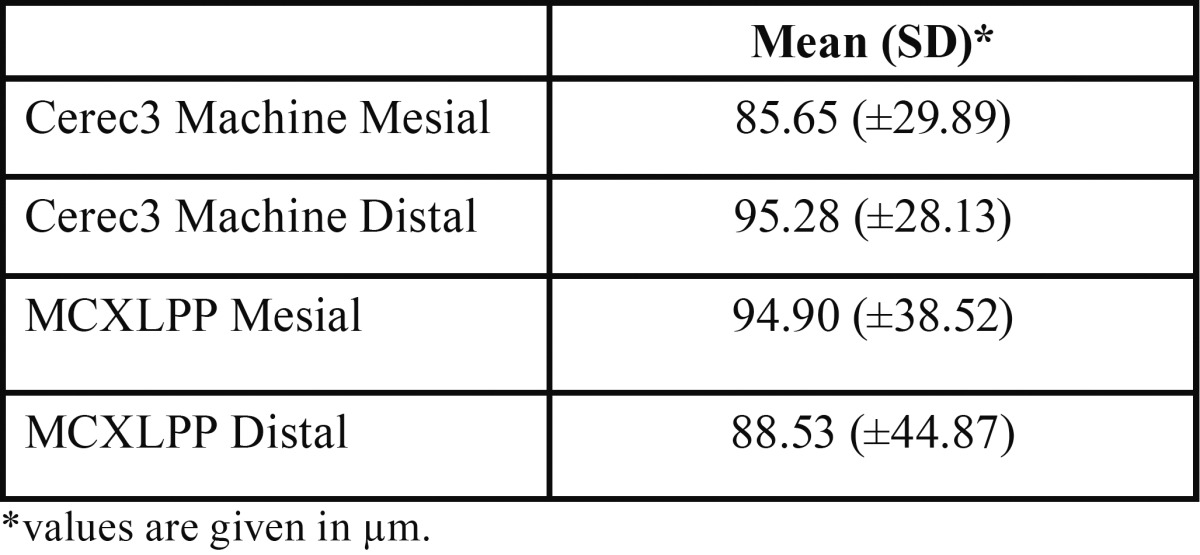
Distribution of mean, standard deviation (SD) and variance for different machines and sites.

For G1 and G2, the analysis associated with individual sites did not show statistically significant differences (*P*<0.05). For each milling unit generation, one-way analysis with Tukey’s post hoc test was made individually to find where the differences among the sites occurred. The *p* value was set at a 0.05 level of significance and is shown in  Table 3, which is the 2-way ANOVA.

**Table 3:**
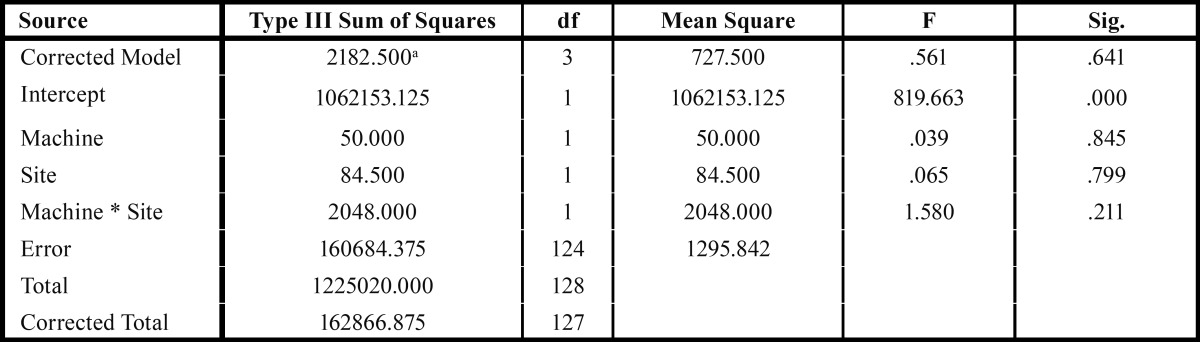
Analyses of Variance (2-way ANOVA).

## Discussion

In the present study, the first null hypothesis was that margin discrepancy of CAD/CAM restorations designed by the same scan unit would be affected by different milling unit generations. For the second null hypothesis, we examined if the different margin sites of the tooth (mesial, and distal) differ in terms of fit, regardless of the milling units used.

We rejected the first null hypothesis because different milling unit generations end up exhibiting similar margin adaptation. Also, we reject the second null hypothesis because different sites on the teeth did not in fact influence the margin adaptation.

The most remarkable findings in the data collected were the similarity in accuracy from both generations of CAD/CAM milling units. During the past three decades, enormous hardware and software improvement on the chair-side acquisition CAD/CAM units have been made. Not differently, the milling units have changed dramatically with main improvements towards speed, noise and better connectivity interface.

The Cerec MC XL premium package is faster and quitter and, it is the current Sirona’s flagship model for chair-side restorations. The Cerec 3 milling unit is currently discontinued, however, still largely used by clinicians because its robust technology, ability to connect to most of the modern Cerec acquisition units, and easy and cheap to repair when maintenance is necessary.

According to Shim *et al.* (15), a fit of a CAD/CAM restoration can be affected by different parameters settings and different software versions. These authors compared the Cerec software 3.8 and 4.2 versions and found that different generation of software can affect the overall quality of the restoration. This study used a Cerec AC with Bluecam unit equipped with the Cerec 4.3 version once this combination has been documented to be a reliable and highly accurate 3-D scan system. Bosh *et al.* (16) used the same scan system in combination with different milling units and concluded that 5-axis milling units presented better overall quality compared with 4-axis machines. Hamza *et al.* (17) found that 5-axis milling unit can actually improve productivity and precision by using the machine’s additional axis. Contrarily, Cho *et al.* (18) concluded that the quality of the final CAD/CAM restoration does not increase with the number of steps and/or bur axes, instead, depending to a greater extent on digitalization, data processing, and production process. The limitation of chair-side CAD/CAM machines containing no more than 4-axis make it difficult to compare this study with ours.

We also observed that distal surfaces of teeth had greater margin discrepancy than the mesial surface. This can be explained by the fact that distal surface of teeth was more difficult to be prepared under indirect vision. Because the mesial surfaces could be easily visualized by direct access, better margin quality was obtained during the preparation phase.

This study did not use natural human extracted teeth because the authors wanted to avoid a large variation due to age, individual structure, and storage time after extraction; consequently, typodont teeth provided a more uniform and standardized abutment. It was also not the objective of this study to test adhesion of teeth to CAD/CAM restorations; instead, crowns were not cemented to the typodont teeth, therefore allowing direct viewing and external measurement. The vertical cervical marginal gap measurement was selected because it is the most common way to quantify the accuracy of fit (15,17).

Using different typodont teeth instead of a single metal die was the selected method in order to introduce variability into the study. This research was not designed to compare different operators; preparations made by a single investigator better simulated a clinical environment and real application of CAD/CAM procedures. Future *in-vitro* and *in-vivo* research in margin discrepancy for chair-side CAD/CAM restorations should be conducted, comparing different brands of available machines with different generations of software and hardware, as well as different CAD/CAM block materials.

Within the limitations of this *in vitro* study, it was concluded that similar margin accuracy was found for both Cerec3 and MCXLPP machines. It seems likely that different generation of Cerec milling units are able to produce clinically acceptable restorations when used in combination with the Cerec BlueCam AC system.
